# Sperm Response to *in vitro* Stress Conditions in Wild and Domestic Species Measured by Functional Variables and ROS Production

**DOI:** 10.3389/fvets.2021.650946

**Published:** 2021-05-24

**Authors:** Emma O'Brien, Pedro García-Casado, Cristina Castaño, Adolfo Toledano-Díaz, Paula Bóveda, Julián Santiago-Moreno

**Affiliations:** ^1^Department of Animal Reproduction, Instituto Nacional de Investigación y Tecnología Agraria y Alimentaria (INIA), Madrid, Spain; ^2^Zoitechlab (Arquimea Group), R&D Department, Madrid, Spain

**Keywords:** spermatozoa, cool-storage, mitochondrial membrane integrity, reactive oxygen species, DNA fragmentation

## Abstract

The domestication process has resulted in profound changes in the reproductive physiology of the animals that might have affected the sperm characteristics and thus their sensitivity to handling and cryopreservation procedures. This work assesses the response of the sperm of domestic and wild ungulates to a cooling storage at 15°C for 20 h followed by incubation at 38.5°C, 5% CO_2_, for 2 h. In addition, this paper examines the most representative sperm traits to assess their responsiveness to these stress conditions. Sperm samples were collected from domestic and their wild ancestor species: ram, mouflon, buck, Iberian ibex, domestic boar, and wild boar. Sperm motility, viability, mitochondrial membrane status, DNA fragmentation, and reactive oxygen species production were evaluated at the beginning of the experiment, after 20 h of refrigeration at 15°C, and, finally, at 2 h of incubation at 38.5°C. Sperm from all domestic species (ram, buck, and domestic boar) suffered more stress than their wild relatives (mouflon, Iberian Ibex, and wild boar). In pigs, the percentage of intact mitochondria was lower in the domestic species compared to wild boar. In sheep, we found a higher reactive oxygen species production in rams, while in goats, the curvilinear velocity was lower in the domestic species. The PCA (principal components analysis) showed that the motility and their kinetic variables were the most represented variables in the principal components of all species, indicating that they are essential biomarkers for evaluating the stress response. Sperm viability was highlighted as a representative variable for evaluating the stress response in domestic boar, mouflon, ram, and ibex.

## Introduction

Many factors affect sperm preserved under chilled conditions. Cold shock is associated with irreversible changes in capacitation, reduction of the sperm motility, and damage to the plasma membrane (1–3). During cooling, sperm cells are exposed to many harmful effects including ionic imbalance, activation of proteases, membrane phase transition, destabilization of the cytoskeleton, and production of free radicals or reactive oxygen species (ROS) (4). Moreover, there is much evidence indicating that sperm cell dysfunction is mainly induced by oxidative stress (5–8). The increase of ROS levels disrupts the mitochondrial and plasma membranes of sperm cells, thus impairing their motility (9). This also causes DNA fragmentation (10), which affects the future sperm fusion with the oocyte.

The sperm sensitivity to cooling and freezing processes may vary among species. These differences may be attributable to variations in plasma membrane compositions (11), for instance, the content of lipids in the bilayer, degree of hydrocarbon chain saturation, cholesterol/phospholipids ratio, and protein/phospholipid ratio (12).

The domestication process in ungulate species has resulted in profound changes in the reproductive physiology (13, 14), such as the endocrine cycles and in the sexual accessory glands activity (15), that might have affected the sperm characteristics and thus their sensitivity to handling and cryopreservation procedures. It is well-known that the animal domestication involved drastic phenotypic changes driven by strong artificial selection to obtain new populations of breeds (16). This selection pressure strongly reduced the initial gene pool. More recently, the selection pressure was increased again via the use of artificial insemination, leading to a few industrial breeds with very high performances, but with low effective population sizes (17). Moreover, just 14 out of the about 30 domesticated mammalian and bird species provide 90% of human food supply from animals. Agriculture has concentrated in the past only on a very small number of breeds worldwide (18). This has led to substantive animal genetic erosion, which could ultimately have affected the resistance capacity of sperm to stress conditions. The questions that arise here are: Are there differences in the sperm cryoresistance between domestic species and their wild ancestors? Has domestication affected the resistance mechanisms of sperm to stress conditions?

To address these questions, we subjected sperm samples from six different species (domestic and their respective wild ancestors) to different incubation conditions (20 h at 15°C and then 2 h at 38.5°C, 5% CO_2_) and we compared their response to these stress conditions. The comparative experimental design included boar (*Sus scrofa domestica*) vs. wild boar (*Sus scrofa*), ram (*Ovis aries*) vs. mouflon (*Ovis musimon*), and buck (*Capra hircus*) vs. ibex (*Capra pyrenaica*). In addition, this paper examines the most representative sperm traits to assess their responsiveness to these stress conditions. To our knowledge, this is the first study where the stress response of sperm is compared between wild and domestic species using diverse techniques to evaluate sperm quality.

## Materials and Methods

### Experimental Animals and Semen Collection

Experimental animals were 31 adult males belonging to six species: ram (*Ovis aries*) aged 5–6 years (*n* = 5), mouflon (*Ovis musimon*) aged 4–8 years (*n* = 6), buck (*Capra hircus*) aged 5–6 years (*n* = 5), Iberian ibex (*Capra pyrenaica*) aged 4–11 years (*n* = 6), domestic boar (*Sus scrofa domestica*) aged 1–2 years (*n* = 5), and wild boar (*Sus scrofa*) aged 1–3 years (*n* = 4).

The rams, bucks, ibexes, and mouflons were housed under natural day length and temperature conditions at the Department of Animal Reproduction of INIA (Madrid, 40°25'N). Boars were housed under 12 h of artificial light and 18–22°C of ambient temperature at La Abadía, Stud Boar from Núcleos Genéticos 21, S. L. (Toledo). Two wild boars were housed at Iberian Kuna Fauna Center (Navas del Rey, Madrid) and two wild boars were captured at the Wildlife Park “El Pardo” (Madrid).

Semen ejaculates were collected from the domestic rams and bucks using an artificial vagina, as previously described Galarza et al. (19). Boar semen collection was performed with the gloved hand method, discarding gel fraction (20). The ejaculated samples from Iberian ibexes, mouflons, and wild boars were obtained by transrectal ultrasound-guided massage of the accessory sex glands (TUMASG), combined with electroejaculation when required (21, 22).

Animals were handled according to procedures approved by the INIA Ethics Committee that specifically approved the design of the current study (reference number PROEX 271/14) and were performed in accordance with the Spanish Policy for Animal Protection (RD53/2013), which conforms to European Union Directive 2010/63/UE regarding the protection of animals used in scientific experiments.

### Experimental Design

All reagents were purchased from Merck KGaA and/or its affiliates (Darmstadt, Germany) and Roche (Basel, Switzerland).

Immediately after sperm collection, each ejaculate was diluted with their species-specific solution (ACROMAX PLUS®, TTG or TCG, depending on the species) at 37°C and transported to the laboratory for initial assessment. Sperm-rich fraction of wild and domestic boar were diluted (1:1 v/v) in ACROMAX PLUS® (ZoitechLab, S.L., Madrid, Spain). Sperm from mouflon and ram were diluted (1:1 v/v) in TTG medium (210.59 mM Tes, 95.75 mM Tris, 10.09 mM glucose, 0.54 mM streptomycin, and 2.14 mM penicillin; 324 mOsm/kg, pH 7.1) (23). Sperm samples of Iberian ibex and buck were diluted (1:1 v/v) in Tris-citric acid-glucose (TCG) medium (313.7 mM Tris, 104.7 mM citric acid, and 30.3 mM glucose; 345 mOsm/kg, pH 6.8) (24).

Diluted samples were centrifuged (900 × *g* for 20 min) and the pellets were re-suspended in their corresponding medium [ACROMAX PLUS®, TTG + 6% egg yolk (EY), or TCG + 6% egg yolk (EY)], to a final concentration of 400 × 10^6^ sperm/ml. Then, samples were subjected to stress: firstly, by refrigerating them at 15°C for 20 h, and then incubating at 38.5°C, 5% CO_2_ for 2 h. The sperm traits of each sample were evaluated at the beginning of the experiment (0H), after 20 h of refrigeration at 15°C (20H 15°C), and, finally, at 2 h of incubation at 38.5°C (20H 15°C + 2H 38.5°C).

### Assessment of Sperm Variables

Sperm viability were assessed using a seminal quality system SQS2® (ZoitechLab, S.L.—ARQUIMEA GROUP, Madrid, Spain) previously validated in our laboratory (see Supplementary Material 1).

Sperm motility analysis was assessed using a computer-aided sperm analysis (CASA) system coupled to a phase contrast microscope (Nikon Eclipse model 50i; Nikon Instruments Europe B.V., Izasa S.A.; negative contrast) and employing a Sperm Class Analyzer (SCA®, Barcelona, Spain) v.4.0. software (Microptic S.L., Barcelona, Spain). Semen was diluted to a concentration of ~40 million sperm/ml (25, 26) and loaded onto a warmed (37°C) 20-μm Leja® 8-chamber slide (Leja Products B.V., Nieuw-Vennep, The Netherlands). The percentage of motile sperm and the percentage showing progressive motility were recorded. A minimum of three fields and 500 sperm tracks were evaluated at a magnification of 100 × for each sample (21). Motility kinetic variables—curvilinear velocity (VCL, μm/s), straight-line velocity (VSL, μm/s), average path velocity (VAP, μm/s), and amplitude of lateral head displacement (ALH, μm)—were also recorded.

DNA integrity was assessed by terminal deoxynucleotidyl transferase dUTP nick end labeling (TUNEL). For this, the kit “*In Situ* Cell Death Detection” (Roche, Basel, Switzerland) was used following the manufacturer's instructions with minor changes (27). Briefly, each sperm sample was diluted to 10 × 10^6^ spermatozoa/ml in 4% paraformaldehyde. Subsequently, 10 μl of this dilution was placed on a glass slide and left to dry. Then, the spermatozoa were permeabilized with 0.1% of Triton X-100 in PBS (137 mM NaCl, 2.7 mM KCl, 8 mM Na_2_HPO_4_, and 2 mM KH_2_PO_4_, pH 7.4). After a wash in PBS, fragmented DNA was nick end-labeled with tetramethylrhodamine-conjugated dUTP by adding 10 μl of the working solution provided by the kit—containing the substrates and the enzyme terminal transferase—on the sample. The reaction was conducted incubating the slides in a humid box for 1 h at 37°C. After a wash with PBS, the nucleus was counterstained with Hoechst at 0.1 mg/ml in PBS for 5 min in the dark. Following an additional wash with PBS, the slides were mounted using Fluoromount (Sigma-Aldrich, MO, USA) and observed under fluorescent microscopy (Eclipse E200, Nikon, Japan). A total of 200 sperm cells per slide were examined using an epifluorescence microscope with a triple band-pass filter (wavelength: 510–560 nm).

Sperm mitochondrial status was assessed using a Mitotracker Green FM® (MITO, Invitrogen M7514), according to Galarza et al. (19) with minor changes. Briefly, samples of 150 μl of semen diluted in TALP Stock (113.94 mM NaCl, 3.08 mM KCl, 0.30 mM NaH_2_PO_4_ H_2_O, 1 mM Na-Lactate, 1.97 mM CaCl_2_ 2H_2_O, 0.50 mM MgCl_2_ 6H_2_O, 10 mM HEPES sodium, and 25 mM NaHCO_3_; 320 mOsm/kg, pH 7.3) to a concentration of 25 × 10^6^ sperm/ml were mixed with 2 μl of MITO (1 mM) and incubated in the dark at 38.5°C for 8 min. After incubation, the samples were transferred to a slide, covered with a cover slip, and examined immediately using an epifluorescence microscope with a triple band-pass filter (excitation: 450 nm, emission: 490 nm). Cells with a high green fluorescent signal in the middle piece were classified as Mitotracker^+^ (Mito+). A total of 200 sperm cells per slide were examined.

Sperm ROS were detected by using CellROX® green (Thermo Fisher Scientific C10444) according to de Castro et al. (28) with minor changes. Samples of 150 μl of semen diluted in TALP Stock to a concentration of 25 × 10^6^ sperm/ml were mixed with CellROX® green (final concentration, 5 μM) and incubated in the dark at 37°C for 30 min. After incubation, samples were washed with TALP Stock ones (10 min at 190 g) and were transferred to a slide, covered with a cover slip, and examined immediately using an epifluorescence microscope with a triple band-pass filter (excitation: 450 nm, emission: 490 nm). CellROX® green is a fluorescent probe that penetrates the cell and, when oxidized by intracellular free radicals, binds to DNA, emitting a more intense green fluorescence. Cells with a green fluorescent signal on the head were classified as low or high CellROX^+^ signal. A total of 200 sperm cells per slide were examined.

The response to stress of each species was illustrated by calculating a stress resistance ratio (SR) for the sperm variables: SR = (value after stress/value before stress) × 100 (see Supplementary Material 2).

### Statistical Analysis

Comparisons of sperm variables at 0H, refrigeration (20H 15°C), and incubation (20H 15°C + 2H 38.5°C) conditions for each species were made using an ANOVA repeated measures. The stress resistance ratio (SR) of domestic species and their wild relatives were compared between groups by the *t* test.

A principal components analysis (PCA) was used to identify which variables best explain the sperm stress response. We performed a PCA of all sperm variables [viability, motility, progressive motility, motility kinetic variables (VCL, VSL, VAP, and ALH), DNA fragmentation, mitochondrial membrane integrity, and low and high levels of ROS] for each six species and treatment period (0H, 20H at 15°C, and 20H at 15°C + 2H at 38.5°C). The optimal number of principal components was determined using the method of cross-validation, where the “optimal number” is defined as the number of principal components that achieves the best goodness of prediction Q^2^X (see Supplementary Material 3).

Data were expressed as means ± SE (standard error of the mean). All statistical calculations were made using Statistica software for Windows v.12 (StatSoft Inc., Tulsa, OK, USA). The significant level was set at *p* < 0.05.

## Results

### Effect of Stress Conditions (20H at 15°C + 2H at 38.5°C) on Sperm Traits of Wild and Domestic Species

For the wild boar sperm, total motility and integrity of mitochondrial membrane decreased (*p* < 0.05), while values of high ROS production increased significantly (*p* < 0.05) (Table 1). For the domestic boar sperm, values of sperm viability, total motility, VSL, VAP, and mitochondrial membrane integrity decreased (*p* < 0.05), while values of both low and high ROS levels increased significantly (*p* < 0.05).

**Table 1:** Wild (*Sus scrofa*) and domestic (*Sus scrofa domestica*) boar sperm quality variables (mean ± SE).

		**Wild boar samples (*****n*** **=** **4)**			**Domestic boar samples (*****n*** **=** **5)**		
**Sperm trait**		**0H**	**20H 15^**°**^C**	**20H 15^**°**^C + 2H 38.5^**°**^C**	**0H**	**20H 15^**°**^C**	**20H 15^**°**^C + 2H 38.5^**°**^C**
Viability (%)		73.7 ± 7.3	73.3 ± 2.7	71.3 ± 4.5	**82.8** **±** **1.4 a**	**73.4** **±** **2.9 ab**	**57.2** **±** **6.9 b**
Motility (%)		**80.6** **±** **8.7 a**	**48.4** **±** **14.9 b**	**45.3** **±** **15.3 b**	**64.1** **±** **14.3 a**	**17.3** **±** **8.9 b**	**20.8** **±** **8.1 b**
Progressive motility (%)		23.4 ± 13.8	4.6 ± 3.6	4.9 ± 3.6	1.3 ± 0.4	0.7 ± 0.5	0.6 ± 0.4
Motility kinetic variables	VCL (μm/s)	49.3 ± 13.3	27.7 ± 4.5	26.8 ± 5.0	21.6 ± 1.2	22.5 ± 2.9	20.2 ± 3.2
	VSL (μm/s)	20.1 ± 4.9	6.4 ± 4.5	5.7 ± 4.3	**7.2** **±** **1.3 a**	**2.3** **±** **1.0 b**	**2.1** **±** **0.5 b**
	VAP (μm/s)	36.6 ± 11.1	11.7 ± 5.2	10.6 ± 5.4	**11.1** **±** **1.3 a**	**6.5** **±** **1.8 ab**	**5.4** **±** **1.1 b**
	ALH (μm)	1.9 ± 0.4	1.5 ± 0.1	1.4 ± 0.2	1.3 ± 0.1	1.35 ± 0.1	1.2 ± 0.1
DNA fragmentation (%)		1.5 ± 0.3	3.7 ± 0.7	3.0 ± 0.9	1.0 ± 0.5	2.8 ± 0.6	3.4 ± 0.7
Mitochondrial membrane integrity (%)		**83.8** **±** **4.6 a**	**75.0** **±** **3.3 ab**	**69.3** **±** **4.8 b**	**66.6** **±** **4.5 a**	**37.0** **±** **8.8 b**	**30.8** **±** **4.2 b**
Low ROS level (%)		34.3 ± 3.2	43.3 ± 2.9	36.3 ± 4.8	**28.6** **±** **2.7 b**	**38.4** **±** **3.2 a**	**41.0** **±** **3.6 a**
High ROS level (%)		**12.5** **±** **3.1 b**	**27.3** **±** **1.1 a**	**28.3** **±** **4.3 a**	**26.0** **±** **7.9 b**	**32.6** **±** **4.7 ab**	**38.0** **±** **5.8 a**

*The sperm variables were evaluated before stress condition (0H), after refrigeration at 15°C for 20 h (20H 15°C), and subsequent incubation at 38.5°C for 2 h (20H 15°C + 2H 38.5°C). Sperm traits: viability, total motility, progressive motility, motility kinetic variables [curvilinear velocity (VCL), straight-line velocity (VSL), average path velocity (VAP), and amplitude of lateral head displacement (ALH)], sperm DNA fragmentation, status of mitochondrial membranes, and oxidative stress level (% of low and high levels of ROS). Means within different letters are significantly different (p < 0.05) between groups (0H, 20H 15°C, and 2H 38.5°C) of each species. Bold values show statistically significant differences between groups*.

For the mouflon sperm, values of ALH and mitochondrial membrane integrity reduced (*p* < 0.05), while percentage of DNA fragmentation and high ROS production increased (*p* < 0.05). Instead, the mitochondrial membrane integrity of ram sperm samples reduced (*p* < 0.05), while the percentage of DNA fragmentation and ROS levels increased (*p* < 0.05) (Table 2).

**Table 2:** Mouflon (*Ovis musimon*) and ram (*Ovis aries*) sperm quality variables (mean ± SE).

		**Mouflon samples (*****n*** **=** **6)**			**Ram samples (*****n*** **=** **5)**		
**Sperm trait**		**0H**	**20H 15^**°**^C**	**20H 15^**°**^C + 2H 38.5^**°**^C**	**0H**	**20H 15^**°**^C**	**20H 15^**°**^C + 2H 38.5^**°**^C**
Viability (%)		63.0 ± 5.3	58.0 ± 4.9	56.0 ± 6.4	70.8 ± 3.8	66.0 ± 5.2	62.4 ± 8.2
Motility (%)		67.9 ± 6.4	62.3 ± 4.5	58.2 ± 6.7	80.3 ± 2.6	87.8 ± 2.9	84.4 ± 5.6
Progressive motility (%)		42.1 ± 9.1	39.8 ± 6.2	33.9 ± 7.1	69.6 ± 4.2	79.2 ± 3.9	77.8 ± 5.9
Motility kinetic variables	VCL (μm/s)	84.1 ± 12.8	87.7 ± 8.4	77.2 ± 9.3	126.2 ± 7.9	131.2 ± 5.3	133.8 ± 5.4
	VSL (μm/s)	43.1 ± 5.9	37.9 ± 4.7	41.4 ± 5.4	74.7 ± 8.5	63.0 ± 1.9	75.3 ± 4.2
	VAP (μm/s)	59.7 ± 7.7	53.7 ± 5.9	56.1 ± 7.6	97.4 ± 9.6	97.2 ± 3.1	102.3 ± 5.0
	ALH (μm)	**2.9** **±** **0.4 ab**	**3.5** **±** **0.3 a**	**2.7** **±** **0.2 b**	3.8 ± 0.2	4.2 ± 0.3	4.2 ± 0.1
DNA fragmentation (%)		**6.8** **±** **1.7 b**	**21.5** **±** **3.7 a**	**25.3** **±** **5.3 a**	**0.6** **±** **0.2 c**	**6.2** **±** **0.7 b**	**15.0** **±** **1.9 a**
Mitochondrial membrane integrity (%)		**81.2** **±** **5.8 a**	**64.3** **±** **7.1 b**	**63.2** **±** **5.2 b**	**83.4** **±** **3.4 a**	**74.4** **±** **4.0 ab**	**66.4** **±** **6.3 b**
Low ROS level (%)		35.3 ± 2.3	35.7 ± 3.8	34.2 ± 4.2	**24.4** **±** **2.4 b**	**40.0** **±** **3.8 a**	**38.0** **±** **1.5 a**
High ROS level (%)		**23.2** **±** **3.9 b**	**37.5** **±** **5.5 a**	**39.0** **±** **4.4 a**	**11.4** **±** **1.6 b**	**40.2** **±** **6.9 a**	**38.0** **±** **2.8 a**

The sperm variables were evaluated before stress condition (0H), after refrigeration at 15°C for 20 h (20H 15°C), and subsequent incubation at 38.5°C for 2 h (20H 15°C + 2H 38.5°C). Sperm traits: viability, total motility, progressive motility, motility kinetic variables [curvilinear velocity (VCL), straight-line velocity (VSL), average path velocity (VAP), and amplitude of lateral head displacement (ALH)], sperm DNA fragmentation, status of mitochondrial membranes, and oxidative stress level (% of low and high levels of ROS). Means within different letters are significantly different (p < 0.05) between groups (0H, 20H 15°C, and 2H 38.5°C) of each species. Bold values show statistically significant differences between groups.

For the Iberian ibex sperm, the values of the mitochondrial membrane integrity decreased significantly (*p* < 0.05), while the percentage of DNA fragmentation increased (*p* < 0.05). For the buck sperm, values of VCL, VSL, and VAP reduced (*p* < 0.05), while levels of ROS production increased (*p* < 0.05) (Table 3).

**Table 3:** Iberian ibex (*Capra pyrenaica*) and buck (*Capra hircus*) sperm quality variables (mean ± SE).

		**Iberian ibex samples (*****n*** **=** **6)**			**Buck samples (*****n*** **=** **5)**		
**Sperm trait**		**0H**	**20H 15^**°**^C**	**20H 15^**°**^C + 2H 38.5^**°**^C**	**0H**	**20H 15^**°**^C**	**20H 15^**°**^C + 2H 38.5^**°**^C**
Viability (%)		64.7 ± 4.4	61.5 ± 4.6	62.5 ± 5.9	60.8 ± 5.8	62.4 ± 2.9	54.5 ± 2.6
Motility (%)		57.7 ± 7.8	52.9 ± 10.4	55.7 ± 8.6	81.9 ± 2.9	80.7 ± 4.5	81.9 ± 1.3
Progressive motility (%)		30.9 ± 11.1	31.2 ± 12.2	31.1 ± 11.8	74.8 ± 4.0	74.3 ± 5.2	72.6 ± 2.0
Motility kinetic variables	VCL (μm/s)	66.7 ± 12.4	62.8 ± 13.4	62.8 ± 12.9	**125.3** **±** **10.3 a**	**99.5** **±** **5.2 b**	**100.7** **±** **6.1 b**
	VSL (μm/s)	44.6 ± 10.9	43.7 ± 12.8	34.8 ± 7.6	**89.9** **±** **6.6 a**	**66.6** **±** **5.5 b**	**67.1** **±** **4.8 b**
	VAP (μm/s)	53.2 ± 12.2	50.2 ± 13.1	43.2 ± 9.5	**110.1** **±** **9.6 a**	**79.3** **±** **5.9 b**	**84.8** **±** **5.8 b**
	ALH (μm)	2.1 ± 0.2	1.9 ± 0.2	2.4 ± 0.4	2.9 ± 0.2	3.1 ± 0.3	2.9 ± 0.1
DNA fragmentation (%)		**7.1** **±** **0.8 b**	**9.7** **±** **1.7 ab**	**10.7** **±** **0.9 a**	0.6 ± 0.2	1.8 ± 0.4	1.4 ± 0.2
Mitochondrial membrane integrity (%)		**72.3** **±** **4.8 a**	**63.0** **±** **4.7 b**	**61.8** **±** **3.5 b**	73.8 ± 7.7	66.6 ± 2.6	61.6 ± 3.4
Low ROS level (%)		39.2 ± 1.6	37.7 ± 3.0	38.0 ± 3.2	19.2 ± 3.3	19.8 ± 2.9	22.0 ± 1.9
High ROS level (%)		17.5 ± 4.4	24.5 ± 3.9	25.0 ± 3.7	**18.8** **±** **2.4 b**	**44.6** **±** **4.2 a**	**44.8** **±** **3.6 a**

*The sperm variables were evaluated before stress condition (0H), after refrigeration at 15°C for 20 h (20H 15°C), and subsequent incubation at 38.5°C for 2 h (20H 15°C + 2H 38.5°C). Sperm traits: viability, total motility, progressive motility, motility kinetic variables [curvilinear velocity (VCL), straight-line velocity (VSL), average path velocity (VAP), and amplitude of lateral head displacement (ALH)], sperm DNA fragmentation, status of mitochondrial membranes, and oxidative stress level (% of low and high levels of ROS). Means within different letters are significantly different (p < 0.05) between groups (0H, 20H 15°C, and 2H 38.5°C) of each species. Bold values show statistically significant differences between groups*.

**Figure 1:**
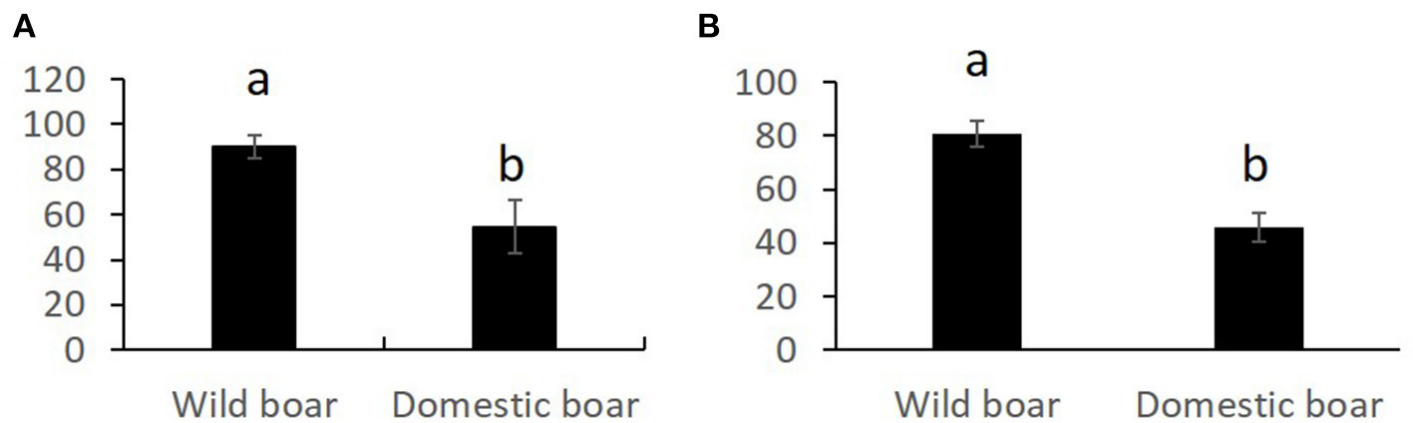
Stress resistance factor (SR) (mean ± SE) for integrity of mitochondrial membrane after 20 h of refrigeration at 15°C **(A)** and after 20 h of refrigeration at 15°C and subsequent incubation at 38°C for 2 h **(B)** in wild and domestic boar sperm samples. Means within different letters are significantly different (*p* < 0.05) between species.

**Figure 2:**
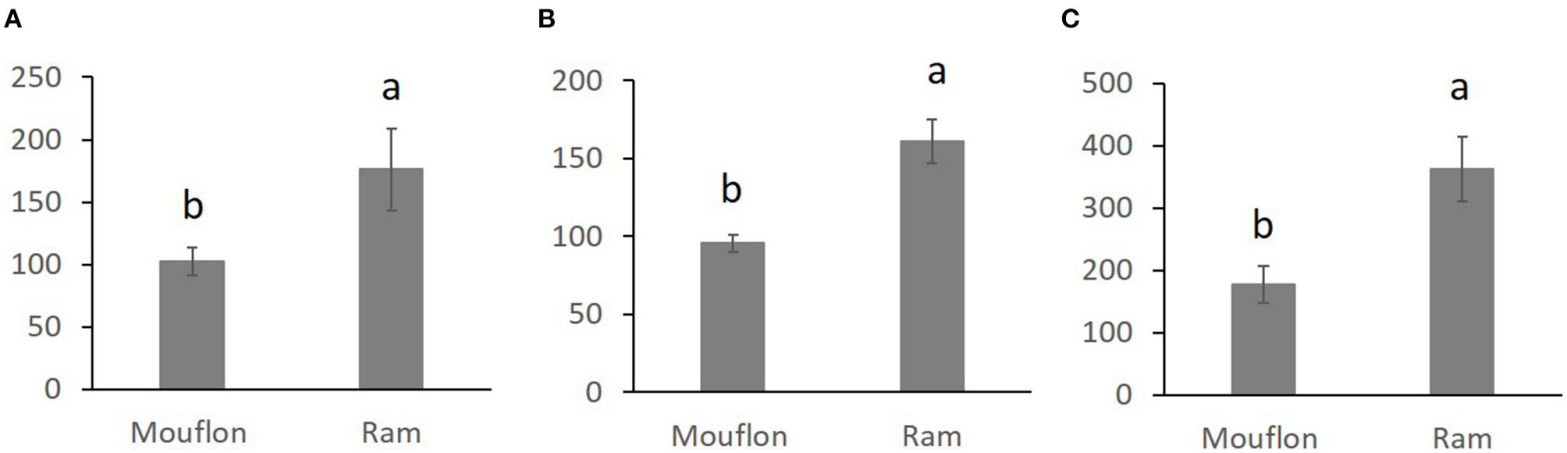
Stress resistance factor (SR) (mean ± SE) for low levels of ROS after refrigeration at 15°C during 20 h **(A)** and after refrigeration at 15°C during 20 h and subsequent incubation at 38.5°C for 2 h **(B)** SR for high levels of ROS after refrigeration at 15°C during 20 h and subsequent incubation at 38.5°C for 2 h **(C)** in mouflon and ram sperm samples. Means within different letters are significantly different (*p* < 0.05) between species.

**Figure 3:**
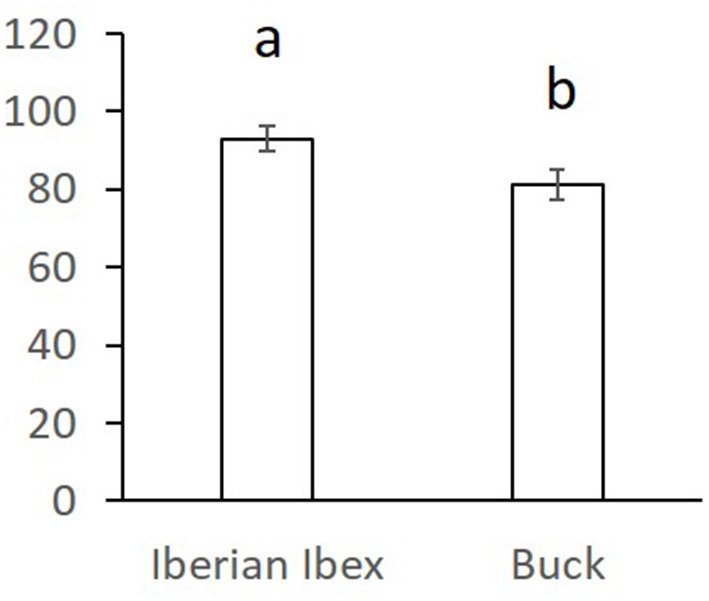
Stress resistance factor (SR) (mean ± SE) for curvilinear velocity (VCL) after refrigeration at 15°C during 20 h and subsequent incubation at 38.5°C for 2 h in ibex and buck sperm samples. Means within different letters are significantly different (*p* < 0.05) between species.

Overall comparisons of the SR, for each sperm variable, between domestic and wild species are shown in the Supplementary Table 3. Only SR values showing significant differences are shown in Figures 1–3. The SR for mitochondrial membrane integrity was lower (*p* < 0.05) in domestic than in wild boar (Figure 1). The SR for ROS production was higher in ram (*p* < 0.05) than in mouflon (Figure 2). In buck, the SR for VCL showed lower values (*p* < 0.05) than in Iberian Ibex (Figure 3).

### The Sperm Variables that Best Explain the Response to Stress

All values for PCA are shown in Supplementary Material 3 (Tables 4–6) for each treatment period: 0H, refrigeration (20H at 15°C), and refrigeration followed by incubation (20H at 15°C + 2H at 38.5°C).

In wild boar, PCA rendered a single principal component (PC) for each period. In the PC for 0H samples (eigenvalue of 7.26), the most represented variables were VAP, VSL, VCL, ALH, total and progressive motility, and high ROS production, while in the PC for refrigerated and incubated samples (eigenvalue 5.85), the most represented variables were VAP, VSL, VCL, ALH, and total and progressive motility. In domestic boar, a single PC was also obtained for each period. In the PC for 0H samples (eigenvalue of 5.30), the most represented variables were VSL, progressive motility, VAP, and high and low ROS production, while in the PC for refrigerated and incubated samples (eigenvalue 6.59), the most represented were those related to motility (and its kinetics) and the sperm viability (Supplementary Table 4).

In mouflon, PCA indicated that two PCs (PC1 and PC2) explained the variability of the data for each period. In PC1 for 0H samples (eigenvalue 5.95), the most represented variables were the total and progressive motility and its kinetics variables (VCL, VSL, VAP, and AHL), while in PC2 (eigenvalue 2.40), the viability and state of the mitochondria were identified as the most represented variables. In PC1 for refrigerated and incubated samples (eigenvalue 5.26) the most represented variables were those related to kinetics motility and low ROS production, while in PC2 (eigenvalue 4.41), the motility, membrane mitochondrial status, and high ROS production were identified as the most represented variables. In ram, a single PC was obtained for each period. In the PC for 0H samples (eigenvalue of 5.78), the most represented variables were ROS production, DNA fragmentation, viability, and VCL, while in the PC for refrigerated and incubated samples (eigenvalue 7.58), the most represented variables were those related to motility (total and progressive motility, VCL, and ALH), viability, DNA fragmentation, and ROS production (Supplementary Table 5).

In Iberian ibex, two PCs (PC1 and PC2) were generated by applying PCA. In PC1 for 0H samples (eigenvalue 7.98), the most represented variables were the total and progressive motility, VCL, VSL, VAP, ALH, mitochondrial status, and high ROS production, while in PC2 (eigenvalue 1.51), the DNA fragmentation was identified as the most represented variables. In PC1 for refrigerated and incubated samples (eigenvalue 7.87), the most represented variables were motility and its kinetics variables, sperm viability and DNA fragmentation, while in PC2 (eigenvalue 1.75), the mitochondrial status and ROS production were identified as the most represented variables. In buck, a single PC was obtained for each period. In the PC for 0H samples (eigenvalue of 6.70), the most represented variables were total and progressive motility, VCL, VSL, VAP, and mitochondrial status. In the PC for refrigerated and incubated samples (eigenvalue 5.16), the most represented variables were those related to kinetics of motility (VCL, VSL, and VAP) and ROS production (Supplementary Table 6).

## Discussion

This work is the first to compare the stress response of sperm from domestic species and their wild ancestors using different semen evaluation techniques. The present findings revealed a species-specific sperm response to stress conditions related to chilling and subsequent incubation. Certainly, the sperm from domestic species appeared to be more sensitive to cooling storage followed by incubation. The percentage of sperm with intact mitochondria in domestic boar was lower than for wild boar. The ROS production was greater in domestic than in wild sheep, while in goats, the VCL was lower in domestic species.

The motility values for the diluted fresh semen in domestic boar were lower than those previously reported for this species: for instance, 88.7% in fresh samples (29) or 90% in samples diluted in ACROMAX® extender (30). After refrigeration and incubation treatments, the worst results for sperm motility, kinetic variables, sperm viability, and membrane mitochondrial status values were observed in porcine species. It is well-known that boar sperm are very susceptible to cold shock (1). The plasma membrane of porcine sperm contains less phosphatidylcholine and more phosphatidylethanolamine and sphingomyelin. In addition, boar sperm exhibits very low content of cholesterol, and therefore, boar sperm is more susceptible to cold damage (31).

Studies aiming at identifying the seminal characteristics of wild boar are scarce (32, 33). The values for sperm motility and viability were similar to those reported in these studies, but to our best knowledge, no studies have been conducted on chilling wild boar sperm. The SR for mitochondrial membrane integrity was higher in wild boar than domestic boar. ROS production increased after chilling and incubation in both wild and domestic boar, but the greatest values of high ROS in wild boar (28.3%) were similar than ROS values before the treatment (0H) in domestic boar (26.0%). Hence, a greater antioxidant capacity of seminal plasma of wild boar should not be ruled out.

The greatest total ROS production (low ROS level + high ROS level) was observed in domestic boar (79%), along with ram (76%), and mouflon (73.2%) samples, suggesting that the balance between ROS production and detoxification by antioxidants was mainly disrupted in these species (11), generating a major oxidative stress. The ram sperm showed the lowest ROS values at 0H, revealing that the ROS production increased considerably during treatment. ROS production strongly increased after refrigeration and incubation in buck sperm, unlike ibex sperm, suggesting that wild goat sperm has a high antioxidant activity like described above for wild boar.

As it was expected, after the cooling period and subsequent incubation, the worst results of DNA fragmentation were observed in sheep samples. In mouflon's samples, the mean percentage of sperm with DNA fragmentation was 25.3%, while in ram, it was 15%. Previous studies in human sperm have already reported that there is a direct relationship between ROS production and DNA damage (34, 35); probably, the high levels of ROS production could be related with the high levels of DNA fragmentation in mouflon and ram samples. Conversely, high levels of fragmented DNA were not detected in boar sperm samples, despite the high production of ROS. Previous studies (30) reported that boar sperm samples diluted in a commercial extender showed very low levels of DNA fragmentation during the preservation (stored at 15°C, during 21 days), in comparison with undiluted semen.

The results confirmed the initial hypothesis that domestication and selection throughout long time ultimately seem to affect the resistance capacity of sperm to stress conditions. Sperm from wild species showed more resistance to stress caused by refrigeration and subsequent incubation than their domestic relatives. Hence, wild species appear to be an excellent model to identify molecular markers related with sperm resistance to stress conditions, such as cold storage (36). The techniques employed in this study allowed us to detect significant differences in some sperm traits; however, other biochemical, and molecular studies would have to be performed in the future, for instance, the characterization of plasma membrane fatty acids (37), the expression of sperm proteins involved in resistance to cold shock (38), the analysis of plasma seminal proteins with antioxidant activities (39), or even the evaluation of differential patterns in RNAm and RNAmi (36).

The PCA measures how well a variable is represented by the principal components and has been used in previous studies of human (40), ibex (41), puma (42), dog (43), turkey (44), and caiman (45) sperm. The aim of PCA is to reduce the dimensionality of a set of variables while retaining the maximum variability. After 22 h of stress, the motility (total or progressive) and their kinetic variables (VCL, VSL, VAP, or ALH) were the most represented variables in all PC of all species. This indicates that motility is an essential biomarker for evaluating the stress response in these species. Our data agree with previous studies performed in Iberian ibex sperm where the sperm motility and motility rate were the most represented variables of the PCA (eigenvalue 2) (41). In addition, sperm viability was the next most represented variable in domestic boar, ram, and Iberian ibex samples, and ROS production was the next most represented variable in mouflon, ram, and buck samples. Despite DNA fragmentation being a very useful variable to evaluate sperm quality and fertility capacity in other species, such as human sperm (46), it was not a substantial contributor to the evaluation of stress resistance in the most studied species, except in the ram and ibex.

In conclusion, motility variables were essential biomarkers for evaluating the stress response in all species. Sperm viability was highlighted as a representative variable for evaluating the stress response in domestic boar, mouflon, ram, and ibexes. The measurement of different sperm functional variables showed that sperm from wild ungulates showed more resistance to stress than sperm from domestic ones.

## Data Availability Statement

The original contributions presented in the study are included in the article/Supplementary Material, further inquiries can be directed to the corresponding authors.

## Ethics Statement

The animal study was reviewed and approved by INIA Ethics Committee.

## Author Contributions

EO'B: sperm collection, sperm analysis, data analysis, and drafted the manuscript. PG-C: discussion of results and drafted the manuscript. CC, AT-D, and PB: sperm collection and sperm analysis. JS-M: experimental design, data analysis, and drafted the manuscript. All authors listed have made a substantial, direct and intellectual contribution to the work, and approved it for publication.

## Conflict of Interest

The authors declare that the research was conducted in the absence of any commercial or financial relationships that could be construed as a potential conflict of interest.
