# Supplementary material for: Sperm Response to in vitro Stress Conditions in Wild and Domestic Species Measured by Functional Variables and ROS Production
Source: Front Vet Sci. 2021 May 24;8:650946. doi: 10.3389/fvets.2021.650946 (PMC8195250; doi:10.3389/fvets.2021.650946)
Supplement: Supplementary file 1 [file Data_Sheet_1.docx]

Supplementary Material

# Assessment of the SQS2® equipment for its application in the evaluation of ruminant sperm

The seminal quality system SQS2® (ZoitechLab, S.L. – ARQUIMEA GROUP, Madrid, Spain) is an automated boar semen analyzer. Their system is based on the cell counting, using a dual fluorescent staining which allows differentiating both live and dead sperm cells. It determines the concentration, percentage of agglutination, viability and three different types of morphoabnormalities (proximal cytoplasmic drop, distal cytoplasmic drop and whiptail) of the sample.

Since there is a similarity in the shape and dimensions of the sperm head between the different ruminant species (the areas of the sperm head of domestic boar, ram, mouflon, Iberian ibex and buck sperm are 33.33 ± 0.05 μm^2^, 35.58 ± 2.59 μm^2^, 39.0 ± 0.10 μm^2^, 30.0 ± 0.09 μm^2^ and 28.20 ± 1.85 μm^2^ respectively, see supplementary table 1) we hypothesized that the equipment could evaluate the sperm functional characteristics with minimal adjustments.

|  | **Length (μm)** | **Width (μm)** | **Area (μm^2^)** | **Perimeter (μm)** | **Reference** |
| --- | --- | --- | --- | --- | --- |
| **Domestic boar** | 8.8 ± 0.0 | 4.44 ± 0.05 | 33.33 ± 0.05 | 27.65 ± 0.04 | Quintero-Moreno et al., 2007 |
| **Ram** | 8.90 ± 0.45 | 4.85 ± 0.20 | 35.58 ± 2.59 | 23.60 ± 0.97 | Flores-Gil et al., 2019 |
| **Mouflon** | 9.30 ± 0.01 | 5.10 ± 0.00 | 39.00 ± 0.10 | 24.0 ± 0.0 | Pradiee et al., 2016 |
| **Buck** | 8.62 ± 0.34 | 3.95 ± 0.17 | 28.20 ± 1.85 | 21.76 ± 0.78 | Flores-Gil et al., 2019 |
| **Iberian ibex** | 8.3 ± 0.02 | 4.4 ± 0.01 | 30.0 ± 0.09 | 21.9 ± 0.04 | Pradiee et al., 2016 |

**Supplementary table 1.** Values of the length, width, area and perimeter of the head of domestic boar, ram, mouflon, buck and Iberian ibex sperm. The values (mean ± SE) for each parameter and the bibliographic references are shown.

Both concentration and viability variables measured with the SQS2® equipment were compared with these obtained by routine methods of our laboratory. The concentration was determined using the SDM 1 photometer (Minitube) for the samples of male bucks and rams, while for mouflons and ibex samples, were carried out with a Neubauer chamber. Sperm viability was determined by propidium iodide staining (PI, Sigma P-4170) as previously described (Soler et al., 2005).

Ten different samples of each species were evaluated (10 replicated of each one) and both methods were compared by means of a statistical analysis (paired t-test) using the STATISTICA® program.

The Supplemental Table 2 shows the global average values of sperm concentration and sperm viability for the all samples of the four species studied (mouflon, ram, Iberian ibex and buck). No statistically significant differences (paired t-test, p<0.05) were observed between the global means of concentration and viability obtained by both methods, for none of the four species analysed.

|  | **Mouflon** |  | **Ram** |  | **Iberian ibex** |  | **Buck** |
| --- | --- | --- | --- | --- | --- | --- | --- |
| Concentration (millions/mL) | 46.49 ± 7.41 |  | 45.07 ± 5.74 |  | 33.19 ± 2.57 |  | 40.37 ± 9.04 |
| Concentration SQS2® (millions/mL) | 44.29 ± 7.22 |  | 42.00 ± 5,26 |  | 32.16 ± 4.77 |  | 35.12 ± 9.26 |
| Viability PI (%) | 64.46 ± 3.27 |  | 56.16 ± 6.81 |  | 57.63 ± 4.65 |  | 61.69 ± 6.78 |
| Viability SQS2® (%) | 62.69 ± 3.74 |  | 53.11 ± 6.56 |  | 58.66 ± 4.16 |  | 57.30 ± 6.30 |

**Supplementary Table 2.** Comparative values (mean ± SE) of sperm concentration (millions of spz/mL) and sperm viability (%) in mouflon, ram, Iberian ibex and buck samples (N = 10). Table shows the values obtained by SQS2® equipment, by photometry (samples of ram and buck) or Neubauer chamber (samples of mouflon and Iberian ibex), and by fluorescence microscopy using PI.

It was concluded that SQS2® equipment was a fast and suitable method for the evaluation of sperm concentrations and sperm viability in small ruminants.

**References:**

Flores-Gil, V.N., Millan de la Blanca, M.G., Velázquez, R., Toledano-Díaz, A., Santiago-Moreno, J., López-Sebastián A. (2020). Influence of testosterone administration at the end of the breeding season on sperm cryoresistance in rams (*Ovis aries*) and bucks (*Capra hircus*). Domest Anim Endocrinol 72, 106425.

Pradiee, J. O'Brien, E., Esteso, M.C., Castaño, C., Toledano-Díaz, A., Lopez-Sebastián, A., Marcos-Beltrán, J.L., Vega. R.S., Guillamón, F.G., Martínez-Nevado, E., Guerra, R., Santiago-Moreno, J. (2016). Effect of shortening the prefreezing equilibration time with glycerol on the quality of chamois (*Rupicapra pyrenaica*), ibex (*Capra* *pyrenaica*), mouflon (*Ovis musimon*) and aoudad (*Ammotragus lervia*) ejaculates. Anim Reprod Sci. 171, 121-8.

Quintero-Moreno, A., González-Villalobos, D., López-Brea, J.J., Esteso, M., Fernández-Santos, M.R., Carvalho-Crociata, J. L., Mejía-Silva, W., León-Atencio, G. (2009). Valoración morfométrica de la cabeza del espermatozoide del cerdo doméstico según su edad. Rev. Cient. (Maracaibo) 19(2), 153-158.

Soler, A.J., Esteso, M.C., Fern andez-Santos, M.R., Garde, J.J. (2005). Characteristics of Iberian red deer (*Cervus elaphus hispanicus*) spermatozoa cryopreserved after storage at 5C in the epididymis for several days. Theriogenology 64, 1503–1517.

# Stress resistance factor (SR)

In order to be able to compare the values obtained between domestic and wild species, we have calculated a factor that allows us to compare the effect of stress, regardless of the initial quality of the different samples. We have called it ‘stress resistance factor’ (SR) and the mathematical formula is:

**SR = (Value after stress / value before stress) x 100**

| **SR** | **Wild boar** | **Domestic boar** | **Mouflon** | **Ram** | **Iberian Ibex** | **Buck** |
| --- | --- | --- | --- | --- | --- | --- |
| **Viability 20H-0H** | 101.78 ± 8.38 | 88.57 ± 2.67 | 92.25 ± 3.74 | 92.89 ± 4.62 | 95.01 ± 2.37 | 106.05 ± 9.54 |
| **Viability**  **20H+2H-0H** | 99.17 ± 10.03 | 69.08 ± 8.23 | 88.85 ± 7.60 | 86.76 ± 8.16 | 96.04 ± 4.50 | 91.93 ± 6.63 |
| **MT 20H-0H** | 57.05 ± 15.10 | 27.93 ± 11.16 | 95.93 ± 10.32 | 109.63 ± 3.99 | 88.87 ± 8.58 | 99.14 ± 6.54 |
| **MT**  **20H+2H-0H** | 51.94 ± 16.38 | 30.21 ± 8.18 | 88.23 ± 10.20 | 105.10 ± 6.35 | 95.25 ± 3.09 | 100.40 ± 3.11 |
| **MP 20H-0H** | 32.92 ± 30.51 | 50.53 ± 38.21 | 142.60 ± 48.56 | 114.92 ± 6.66 | 100.26 ± 32.96 | 100.76 ± 8.83 |
| **MP 20H+2H-0H** | 34.91 ± 30.61 | 33.13 ± 20.30 | 103.05 ± 24.99 | 111.94 ± 6.40 | 97.61 ± 6.62 | 98.35 ± 6.31 |
| **VCL 20H-0H** | 65.05 ± 13.21 | 106.87 ± 17.79 | 113.25 ± 12.83 | 106.32 ± 10.18 | 94.07 ± 11.54 | 80.39 ± 3.66 |
| **VCL 20H+2H-0H** | 61.54 ± 12.10 | 94.54 ± 15.44 | 96.63 ± 8.21 | 108.08 ± 9.26 | **92.85 ± 3.25** | **81.24 ± 3.75** |
| **VSL 20H-0H** | 33.91 ± 21.67 | 33.85 ± 13.21 | 96.76 ± 14.4 | 87.55 ± 7.20 | 97.20 ± 19.42 | 74.29 ± 4.44 |
| **VSL 20H+2H-0H** | 29.15 ± 20.82 | 29.64 ± 5.25 | 99.66 ± 8.92 | 106.06 ± 12.91 | 81.11 ± 5.89 | 75.22 ± 4.90 |
| **VAP 20H-0H** | 38.99 ± 15.56 | 60.07 ± 16.08 | 96.14 ± 11.52 | 102.89 ± 8.72 | 94.61 ± 16.98 | 72.76 ± 4.04 |
| **VAP 20H+2H-0H** | 33.57 ± 15.71 | 49.40 ± 10.52 | 95.86 ± 7.88 | 110.12 ± 14.00 | 82.43 ± 4.63 | 77.94 ± 4.42 |
| **ALH 20H-0H** | 82.13 ± 11.44 | 107.20 ± 14.33 | 125.32 ± 11.67 | 110.53 ± 10.44 | 91.81 ± 4.49 | 106.22 ± 9.71 |
| **ALH 20H+2H-0H** | 74.56 ± 10.78 | 95.87 ± 14.53 | 98.63 ± 9.07 | 111.50 ± 6.96 | 109.51 ± 7.84 | 101.28 ± 6.21 |
| **Tunel 20H-0H** | 283.33 ± 47.32 | 113.33 ± 74.23 | 563.10 ± 270.26 | 360.00 ± 156.84 | 134.88 ± 17.16 | 120.00 ± 58.30 |
| **Tunel 20H+2H-0H** | 266.66 ± 94.64 | 113.33 ± 59.25 | 521.59 ± 161.04 | 860.00 ± 384.18 | 155.29 ± 15.47 | 80.00 ± 37.41 |
| **Mito 20H-0H** | **90.17 ± 5.28** | **54.58 ± 11.91** | 79.15 ± 6.33 | 89.07 ± 2.20 | 86.96 ± 2.29 | 93.19 ± 7.19 |
| **Mito 20H+2H-0H** | **82.77 ± 3.98** | **45.84 ± 5.62** | 78.47 ± 4.99 | 79.46 ± 6.11 | 86.30 ± 4.53 | 85.96 ± 6.62 |
| **Low ROS 20H-0H** | 127.57 ± 5.23 | 137.73 ± 13.89 | **101.98 ± 11.12** | **176.30 ± 32.86** | 96.36 ± 7.46 | 110.36 ± 18.12 |
| **Low 20H+2H-0H** | 110.03 ± 20.77 | 144.79 ± 10.97 | **95.34 ± 5.45** | **160.97 ± 14.23** | 98.96 ± 10.64 | 126.83 ± 22.26 |
| **High ROS 20H-0H** | 280.89 ± 87.17 | 157.19 ± 26.72 | **177.15 ± 29.28** | **362.11 ± 51.97** | 171.76 ± 33.66 | 242.51 ± 17.32 |
| **High 20H+2H-0H** | 301.43 ± 121.46 | 184.13 ± 35.49 | 191.00 ± 33.56 | 385.73 ± 94.34 | 185.01 ± 41.10 | 259.14 ± 43.39 |

We have calculated the SR for each sperm variables at different times of the experimental period: after 20 h of refrigeration at 15° C (SR 20H-0H) treatment, and after refrigeration and subsequent incubation at 38°C for 2 h (SR 20H+2H-0H) (see Supplementary Table 3).

**Supplementary Table 3.** Stress resistance factor (SR) (mean ± SE) for each sperm variable and species through the experimental period: after 20 h refrigeration (20H-0H); after 20 h refrigeration + 2 h incubation (20H+2H-0H). The factor SR was calculated for the following sperm variables: viability, total motility (MT), progressive motility MP), motility kinetic variables; curvilinear velocity (VCL), straight-line velocity (VSL), average path velocity (VAP), amplitude of lateral head displacement (ALH), sperm DNA fragmentation (TUNEL), mitochondrial membrane integrity (Mito) and oxidative stress levels (% of low and high ROS production). Values less than 100% mean that the variable decreased after the stress, and values greater than 100 that it increased.

# Principal Components Analysis

The aim of PCA is to reduce the dimensionality of a set of variables while retaining the maximum variability in terms of the variance-covariance structure. The variable importance is a useful quantity in PCA analysis. It measures how well a variable is represented by the principal components.

As one of our objectives is to identify if there is a sperm variable that best describes the effect of stress, we performed a PCA analysis of the eleven sperm variables (viability, motility, progressive motility, motility kinetic variables -VCL, VSL, VAP and ALH-, DNA fragmentation, mitochondrial membrane integrity and low and high levels of ROS) for each one of the six species for each treatment period (at 0H, refrigeration -20H at 15ºC- and subsequent incubation -2H at 38.5°C-). All values for PCA analysis are shown in Supplementary Tables 4 to 6.

|  |  | **0h** | | **Refrigeration at 15ºC for 20 h** | | **Refrigeration at 15ºC for 20 h and incubation at 38.5° for 2 h** | |
| --- | --- | --- | --- | --- | --- | --- | --- |
|  |  | **Wild boar** | **Domestic Boar** | **Wild boar** | **Domestic Boar** | **Wild boar** | **Domestic Boar** |
|  |  | **PC** | **PC** | **PC** | **PC** | **PC** | **PC** |
| **Sperm variables** | | Factor loadings | Factor loadings | Factor loadings | Factor loadings | Factor loadings | Factor loadings |
| Viability | | 0.831128 | -0.005679 | 0.440498 | -0.690957 | 0.268134 | **-0.823085** |
| Motility | | **0.836335** | 0.793757 | 0.781864 | **0.976575** | **0.807701** | **0.936784** |
| Progressive motility | | **0.961662** | **0.947079** | **0.970501** | **0.975813** | **0.973768** | **0.802647** |
| Motility kinetic variables | VCL | **0.988295** | 0.190780 | **0.992699** | 0.594472 | **0.908663** | **0.867343** |
|  | VSL | **0.985341** | **0.972457** | **0.942273** | **0.811535** | **0.938741** | **0.886470** |
|  | VAP | **0.999790** | **0.863046** | **0.963904** | **0.856060** | **0.960596** | **0.919249** |
|  | ALH | **0.971963** | -0.005679 | **0.911964** | 0.382187 | **0.850845** | **0.858405** |
| DNA fragmentation | | 0.114836 | -0.279275 | **-0.994259** | **-0.790036** | -0.506165 | 0.047288 |
| Mitochondrial membrane integrity | | 0.435642 | 0.717895 | 0.167457 | **0.864925** | -0.114052 | 0.563477 |
| High ROS levels | | **-0.859245** | **0.853634** | **0.880287** | 0.658212 | -0.390586 | 0.632295 |
| Low ROS levels | | 0.346973 | **-0.850921** | **-0.866455** | 0.455772 | 0.636577 | -0.742613 |
| R^2^X | | 0.66 | 0.48 | 0.72 | 0.57 | 0.53 | 0.59 |
| Eigenvalues | | 7.26 | 5.30 | 7.92 | 6.28 | 5.85 | 6.59 |
| Variance explained (%) | | 66.07 | 48.18 | 72.03 | 57.15 | 53.20 | 59.91 |

**Supplementary Table 4:** Principal components (PC) results at 0h, chilled at 15ºC for 20 h and refrigeration at 15°C during 20 h and subsequent incubation at 38.5°C for 2 h of wild boar and domestic boar sperm. The values of loading factor of each sperm variable, the R^2^X statistics, the eigenvalues and variance explained (%) are shown. The sperm variables that best explain each PC are indicated in bold type.

|  |  | **0h** | | | **Refrigeration at 15ºC for 20 h** | | | **Refrigeration at 15ºC for 20 h**  **and incubation at 38.5° for 2 h** | | |
| --- | --- | --- | --- | --- | --- | --- | --- | --- | --- | --- |
|  |  | **Mouflon** | | **Ram** | **Mouflon** | | **Ram** | **Mouflon** | | **Ram** |
|  |  | **PC1** | **PC2** | **PC1** | **PC1** | **PC2** | **PC1** | **PC1** | **PC2** | **PC1** |
| **Sperm variables** | | Factor loadings | | Factor loadings | Factor loadings | | Factor loadings | Factor loadings | | Factor loadings |
| Viability | | 0.349656 | **0.913881** | **0.755475** | -0.363832 | **-0.713841** | **0.821842** | 0.773556 | 0.633839 | **0.849165** |
| Motility | | **0.889282** | 0.286025 | 0.508616 | 0.404589 | **-0.898625** | **0.918234** | 0.300283 | **0.935658** | **0.943673** |
| Progressive motility | | **0.984873** | 0.140123 | 0.689894 | **0.783380** | -0.604808 | **0.923174** | -0.244531 | **0.940327** | **0.956902** |
| Motility kinetic variables | VCL | **0.963684** | -0.181120 | **0.880774** | **0.964902** | 0.065768 | 0.715982 | **-0.903077** | 0.413664 | **0.940558** |
|  | VSL | **0.993184** | -0.109452 | 0.582313 | **0.913146** | -0.127365 | -0.520360 | **-0.831605** | 0.470780 | 0.505750 |
|  | VAP | **0.994421** | -0.083705 | **0.712078** | **0.981856** | -0.129849 | 0.141549 | **-0.854914** | 0.507028 | 0.754061 |
|  | ALH | **0.912995** | -0.222625 | 0.697519 | **0.774655** | 0.289898 | **0.779750** | **-0.907638** | 0.323827 | **0.814792** |
| DNA fragmentation | | 0.231266 | -0.725319 | **-0.855110** | 0.274546 | 0.453309 | 0.687023 | -0.319436 | -0.634104 | **-0.891551** |
| Mitochondrial membrane integrity | | 0.175324 | **0.855984** | 0.332268 | -0.637509 | **-0.763998** | **0.973074** | 0.632729 | **0.722991** | **0.843888** |
| High ROS levels | | 0.357105 | -0.031629 | **0.800841** | 0.147639 | -0.706041 | -0.447949 | 0.504792 | **0.752792** | **0.776745** |
| Low ROS levels | | 0.346225 | -0.326953 | **0.944943** | 0.055629 | 0.469997 | -0.302281 | **-0.832940** | 0.106724 | -0.758116 |
| R^2^X | | 0.54 | 0.21 | 0.52 | 0.43 | 0.3 | 0.49 | 0.47 | 0.4 | 0.68 |
| Eigenvalues | | 5.95 | 2.4 | 5.78 | 4.74 | 3.31 | 5.49 | 5.26 | 4.41 | 7.58 |
| Variance explained (%) | | 54.11 | 21.86 | 52.63 | 43.14 | 30.11 | 49.93 | 47.82 | 40.17 | 68.95 |
| Variance explained accumulated (%) | |  | 75.97 | 52.63 |  | 73.25 | 49.93 |  | 88.00 | 68.95 |

**Supplementary Table 5:** Principal components (PC) results at 0 h, chilled at 15ºC for 20 h and refrigeration at 15°C during 20 h and subsequent incubation at 38.5°C for 2 h of mouflon and ram sperm. The values of loading factor of each sperm variable, the R^2^X statistics, the eigenvalues, variance explained (%) and variance explained accumulated (%) are shown. The variables that best explain each PC are indicated in bold type.

|  |  | **0h** | | | **Refrigeration at 15ºC for 20 h** | | | | **Refrigeration at 15ºC for 20 h and incubation at 38.5°C for 2 h** | | |
| --- | --- | --- | --- | --- | --- | --- | --- | --- | --- | --- | --- |
|  |  | **Iberian Ibex** | | **Buck** | **Iberian Ibex** | | | **Buck** | **Iberian Ibex** | | **Buck** |
|  |  | **PC1** | **PC2** | **PC1** | **PC1** | **PC2** | | **PC1** | **PC1** | **PC2** | **PC1** |
| **Sperm variables** | | Factor loadings | | Factor loadings | Factor loadings | | | Factor loadings | Factor loadings | | Factor loadings |
| Viability | | 0.640530 | 0.537876 | 0.768812 | 0.766397 | | 0.239524 | **0.852521** | **0.791109** | 0.433092 | -0.456260 |
| Motility | | **0.978621** | 0.149669 | **0.968578** | **0.963943** | | 0.007942 | **0.833622** | **0.957922** | -0.051868 | 0.133644 |
| Progressive motility | | **0.989493** | 0.019971 | **0.846227** | **0.980652** | | -0.128400 | **0.793480** | **0.954791** | -0.296794 | 0.506349 |
| Motility kinetic variables | VCL | **0.983814** | -0.034837 | **-0.864738** | **0.970941** | | -0.225852 | 0.357208 | **0.943380** | -0.329641 | **0.863383** |
|  | VSL | **0.987361** | -0.006539 | **-0.827150** | **0.928242** | | -0.353714 | 0.171012 | **0.874382** | -0.456280 | **0.993844** |
|  | VAP | **0.983168** | -0.014252 | **-0.839282** | **0.936808** | | -0.329897 | 0.274208 | **0.901066** | -0.410011 | **0.952976** |
|  | ALH | **0.886477** | -0.126105 | -0.581131 | **0.893976** | | 0.388904 | 0.154296 | **0.964120** | -0.161192 | 0.444883 |
| DNA fragmentation | | 0.728785 | **0.646916** | 0.798873 | 0.117362 | | 0.530961 | **0.913147** | **0.755755** | 0.458035 | -0.414095 |
| Mitochondrial membrane integrity | | **0.929511** | -0.304255 | **0.889132** | **0.823070** | | 0.322819 | 0.676607 | 0.738484 | **0.602052** | -0.618686 |
| High ROS levels | | **0.725093** | -0.616669 | -0.695327 | 0.553716 | | **0.581175** | **-0.932572** | 0.703630 | 0.045738 | **-0.809305** |
| Low ROS levels | | -0.159111 | 0.538850 | -0.269246 | 0.240978 | | **-0.855402** | -0.501072 | 0.640069 | **0.628500** | **0.797310** |
| R^2^X | | 0.72 | 0.13 | 0.60 | 0.63 | | 0.17 | 0.42 | 0.71 | 0.15 | 0.46 |
| Eigenvalues | | 7.98 | 1.51 | 6.70 | 7.01 | | 1.96 | 4.71 | 7.87 | 1.75 | 5.16 |
| Variance explained (%) | | 72.62 | 13.73 | 60.94 | 63.77 | | 17.87 | 42.90 | 71.56 | 15.98 | 46.96 |
| Variance explained accumulated (%) | |  | 86.36 | 60.94 |  | | 81.64 | 42.90 |  | 87.55 | 46.96 |

**Supplementary Table 6:** Principal components (PC) results at 0 h, chilled at 15ºC for 20 h and refrigeration at 15°C during 20 h and subsequent incubation at 38.5°C for 2 h of Iberian Ibex and buck sperm. The values of loading factor of each sperm variable, the R^2^X statistics, the eigenvalues, variance explained (%) and variance explained accumulated (%) are shown. The variables that best explain each PC are indicated in bold type.
